# Alternative treatment of serious and mild *Pasteurella multocida* infection in New Zealand White rabbits

**DOI:** 10.1186/s12917-014-0276-6

**Published:** 2014-11-25

**Authors:** Orsolya Palócz, János Gál, Paul Clayton, Zoltán Dinya, Zoltán Somogyi, Csaba Juhász, György Csikó

**Affiliations:** Department of Pharmacology and Toxicology, Faculty of Veterinary Science, Szent István University, István u. 2, Budapest, 1078 Hungary; Department of Exotic Animal and Wildlife Medicine, Faculty of Veterinary Science, Szent István University, István u. 2, Budapest, 1078 Hungary; Institute for Food, Brain and Behaviour, 106-108. Cowley Road, Oxford, OX41JE, UK; Flavon Group Hungary, Veres Péter u. 19, Debrecen, 4033 Hungary; Immitec Nutrition AB, Farmannsveien 18-22, Tønsberg, 3111 Norway

**Keywords:** *Pasteurella multocida*, β-glucan, Rabbit

## Abstract

**Background:**

*Pasteurella multocida* causes numerous economically relevant diseases in livestock including rabbits. Immunisation is only variably effective. Prophylactic antibiotics are used in some species but are contra-indicated in rabbits, due to their adverse effects on the rabbit microbiota. There is therefore a substantial need for alternative forms of infection control in rabbits; we investigated the effect of oral β-glucan on *P. multocida* infection in this species.

**Results:**

Thirthy-five New Zealand White rabbits were randomly divided into five groups of seven animals. Three groups were inoculated with *Pasteurella multocida* intranasally (in.), a physiologically appropriate challenge which reproduces naturally acquired infection, and received either (1–3), (1–6) β-glucans or placebo. Four other groups were inoculated both in. and intramuscularly (im.), representing a supra-physiological challenge, and received either (1–3), (1–6) β-glucans, antibiotic or placebo. β-glucans given prophylactically were highly effective in protecting against physiological (in.) bacterial challenge. They were less effective in protecting against supra-physiological bacterial challenge (in. and im.), although they extended survival times. This latter finding has practical relevance to breeders as it extends the window in which heavily infected and symptomatic animals can be salvaged with antibiotics.

**Conclusions:**

In our study, (1–3), (1–6) β-glucans were highly effective in protecting against a model of naturally acquired *P. multocida* infection and extended survival times in the supra-physiological model. Enrofloxacin was effective in protecting against supra-physiological infection. We are currently reviewing the use of combined prophylaxis.

**Electronic supplementary material:**

The online version of this article (doi:10.1186/s12917-014-0276-6) contains supplementary material, which is available to authorized users.

## Background

In infection control, rabbits are important target animals. They are domestic pets, livestock animals in increasing demand for meat and fur production, and a widely used experimental species for human drug research.

Since the European Union (EU) and Regulation (EC) 1831/2003 banned the use of antibiotics as feed additives in 2006, concerted efforts have been made to find alternative prophylactics. To date none of the investigated supplements has shown adequate effectiveness, leading to reduced feed conversion rates and increased per-head veterinary and other costs [1].

In rabbit production, antibiotics are still commonly used under veterinary prescription during the growth period (weaning – 56/60 days) to prevent post-weaning enteric disorders. However, regulatory changes affecting rabbit production similar to those applied to poultry are expected shortly [2]. As much rabbit production is small to medium scale, many producers will be unable to afford to make substantial changes in husbandry practice. Alternative prophylactics are therefore urgently needed.

*Pasteurella multocida*, a virulent and readily transmitted coccobacillus, is one of the most significant bacterial diseases of rabbits and causes considerable economic losses in large production units world-wide [3,4]. Clinical signs in rabbits include rhinitis with purulent nasal discharge, pneumonia, otitis media, pyometra, orchitis, abscesses, oculoconjunctivitis, and septicaemia [5,6]; although infection may also be asymptomatic [7]. Of the 5 serogroups, serogroup A and to a lesser extent serogroup D strains are considered to be causative agents of pasteurellosis in rabbits [8]. Rabbits can become infected with *P. multocida* immediately after birth, and the prevalence of colonization increases with age until about 5 months. Most adult rabbits are believed to be infected with *P. multocida*.

Endemic infection, therefore, remains a source of considerable potential loss. Control measures such as enhanced husbandry and the culling of symptomatic animals reduce overall morbidity and mortality, but impose significant costs. Vaccines targetting *P. multocida* reduce infection but do not give complete protection under field conditions [9]. Antibiotics are due to be phased out, due to such concerns as antibiotic traces in animal products impacting on human health [10]. In any case, and despite initial reports of multiple antibiotic sensitivity, [11,12], the spread of resistance [13] and the hypersensitivity of rabbits to many antibacterial agents precludes the aggressive use of antibiotics in this species [14].

Alternative to antibiotics include probiotics, prebiotics and a number of phytonutrients, none of which is very effective. To this list we must now add the β-glucans, cell wall constituents of bacteria, fungi and plants. The (1–3), (1–6) fungal β-glucans in particular are recognized by mammalian cells as pathogen-associated molecular patterns and thus act as biological response modifiers. This recognition plays an important role in host defense and presents specific opportunities for clinical modulation of the host innate immune response. Experimental and clinical results show that the (1–3), (1–6) β-glucans act as broad-spectrum enhancers of host defense mechanisms, positively influencing the immunological response of mammals including humans to bacterial, viral, and fungal infections [15].

The aim of this present study was to observe and describe the effect of a (1–3), (1–6) β-glucan dietary supplement on physiological and a supra-physiological *P. multocida* infection in rabbits.

## Methods

### Bacterial isolates and growth conditions

*Pasteurella* isolates were stored in a solution of 30% glycerol at −80°C. Isolates were grown in tryptic soy broth at 36°C.

For the isolation of *P. multocida* from clinical samples, swabs were streaked onto blood agar plates and incubated at 36°C for 24 hours.

### Rabbits, housing and treatments

Thirty-five, clinically healthy, seven week old, 20 female and 15 male New Zealand White rabbits (S & K Lap Nyúltenyésztő Kft., Kartal, Hungary) were used in this study. The animals were housed in stainless steel cages (40×60×80 cm), two rabbits per cage in the animal housing facility of the Department of Pharmacology and Toxicology, acclimated at 20 ± 2°C on a 12 h light 12 h dark schedule. One hundred grams of commercial pellet feed was provided daily, water was available *ad libitum*. A microchip was implanted sub-dermally (cervical region) in each animal for validated identification. The rabbits (mean weight 1105 ± 87 g) were randomly divided into 5 groups (7 rabbits/group) comprising 1 control group and 4 experimental groups: positive controls, antibiotic-treated, low dose β-glucan (5 mg/kg bw.) and high dose β-glucan (50 mg/kg bw.) (Table 1).

**Table 1:** **Experimental schedule**

**Group name**	**Days 8–** **14 at 8 am**	**Days 15–** **21 at 8 am**
Control	0.5% methylcellulose 1 ml/kg	0.5% methylcellulose 1 ml/kg
Positive control	0.5% methylcellulose 1 ml/kg	0.5% methylcellulose 1 ml/kg
Antibiotic	0.5% methylcellulose 1 ml/kg	1% enrofloxacin in 0.5% methylcellulose
Small dose beta-glucan	5 mg/kg beta-glucan in 0.5% methylcellulose	5 mg/kg beta-glucan in 0.5% methylcellulose
High dose beta-glucan	50 mg/kg beta-glucan in 0.5% methylcellulose	50 mg/kg beta-glucan in 0.5% methylcellulose

The substances were administered to the rabbits in the concentrations above, according to the time schedule.

The experimental schedule included acclimatization for 7 days, pre-treatment for 14 days, inoculation, and subsequent post-treatment for 7 days. During the pre-treatment phase from day 8 to 21, β-glucan was delivered in aqueous solution via probe, made up with 0.5% w/v methylcellulose. Animals in the control and antibiotic groups received pure methylcellulose solution. After challenge with *P. multocida* (see below), in the control groups the daily single administration of placebo was continued. On the day of challenge and thereafter, animals in the antibiotic group daily received enrofloxacin 10 mg/kg bw., diluted ten times in 0.5% methylcellulose.

Bakers yeast β-glucan (Immivet® 3–6 Dispersible Powder) was supplied by Biothera Company (Eagan, MN 55121, USA).

*Methylcellulosum* USP; Molar Chemicals Ltd, Budapest, Hungary.

Enrofloxacin: Neoflox® 10% internal solution; Tolnagro Ltd., Szekszárd, Hungary.

### Bacterial challenge and subsequent monitoring

Bacterial challenges were administered on Day 15. In the three physiological model groups, animals were given in. 0.5 ml physiological saline containing approximately 10^8^ CFU of a cocktail of ten lapine isolates of *P. multocida*. In the four supra-physiological model groups, animals received 0.5 ml of the same bacterial suspension both in. and im. Rectal temperature and clinical status of all rabbits were monitored twice daily. The clinical signs were scored as follows: breathing sound 0 – normal, 1 – respiratory murmur; nasal discharge 0 – absent, 1 – present; conjunctivitis 0 – absent, 1 – present; attitude 0 – active, 1 – depressed; appetite 0 – normal, 1 – decreased. A total clinical score was calculated for each individual rabbit (0–5). Challenged rabbits in the terminal stage of the disease and those which survived until 7 days pi. were euthanized by sodium pentobarbital overdose (100 mg/kg bw. ip., Euthasol® 40% injection, Produlab Pharma B.V., Netherlands).

### Pathology

All challenged and control rabbits were examined for gross and histological lesions. Lungs, liver, spleen, kidneys and nasal mucosa were histologically examined in all rabbits. The samples were fixed in 8% neutral buffered formalin for 24 h and embedded in paraffin wax. Sections (3–4 μm) were cut, stained with hematoxylin and eosin, and examined by light microscopy.

### PCR analyses and gel electrophoresis

Total DNA of bacterial strains was isolated with E.Z.N.A. bacterial DNS kit (OMEGA Bio-Tek, Norcross, USA) according to the manufacturer’s recommendations.

The identity of isolates was confirmed by PCR. PCR was performed using 5PRIME HotMasterMix (5PRIME GmbH, Hamburg, Germany) on the MiniOpticon System (BioRad). For each PCR reaction, 2.5 μl DNA was added directly to a PCR reaction mixture, set to a final volume of 25 μl, containing × 1 concentrated HotMasterMix and 0.2 μM of the appropriate primers. The DNA-sequences of oligonucleotide primers are listed in Table 2. The thermal profile for all reactions was 2 min at 95°C, then 30 cycles of 30 s at 95°C, 30 s at 60°C and 30 s at 72°C. The PCR was terminated with a final extension at 72°C for 5 min.

**Table 2:** **Primer sequences for identification and detection of the virulence associated genes in** ***P. multocida*** **strains**

**Gene**	**Primer sequence**	**Accession number**	**Reference**
*kmt1*	F - ATCCGCTATTTACCCAGTGG	[GenBank:NC_017027]	[21]
	R - GCTGTAAACGAACTCGCCAC		
*hyaC*-*hyaD*	F - TGCCAAAATCGCAGTCAG	[GenBank:NZ_CM002276]	[22]
	R - TTGCCATCATTGTCAGTG		
*pfhA*	F - AGCTGATCAAGTGGTGAAC	[GenBank:NC_016808]	This study
	R - TGGTACATTGGTGAATGCTG		
*nanH*	F - GAATATTTGGGCGGCAACA	[GenBank:NC_017764]	[23]
	R - TTCTCGCCCTGTCATCACT		
*hgbA*	F - TGGCGGATAGTCATCAAG	[GenBank:NZ_CM002276]	[23]
	R - CCAAAGAACCACTACCCA		
*ompH*	F - CGCGTATGAAGGTTTAGGT	[GenBank:NZ_CM001580]	[23]
	R - TTTAGATTGTGCGTAGTCAAC		
*ptfA*	F - TCCACTCGTTGTGGCATTCA	[GenBank:NC_017027]	This study
	R - AGAAACACCTTGAGCTGCGT		

The PCR amplicons were separated by electrophoresis in 2% agarose gel, the resulting bands were visualised and scanned by the InGenius LHR Gel Documentation and Analysis System (Syngene).

### Statistical analyses

Statistical analyses were performed by Statistica 12 software (Statsoft, Tulsa, USA). Differences between means were evaluated by one-way analysis of variance (ANOVA) followed by a post hoc comparison using Fisher’s least significant difference (LSD) test. Survival curves were calculated using the Kaplan-Meier method, the curves were compared using the Cox’s F test. Statistical significance was set at p <0.05.

### Ethical approval

The experiment was conducted according to approved laboratory animal experimentation ethics regarding to the national and European law, compatible with the conditions set up by the code of practice for the care and use of animals for experimental purpose. The study was authorised by the Local Institutional Animal Care Committee (Munkahelyi Állatjóléti Bizottság, MÁB) (no. 51/2013).

## Results

**Figure 1:**
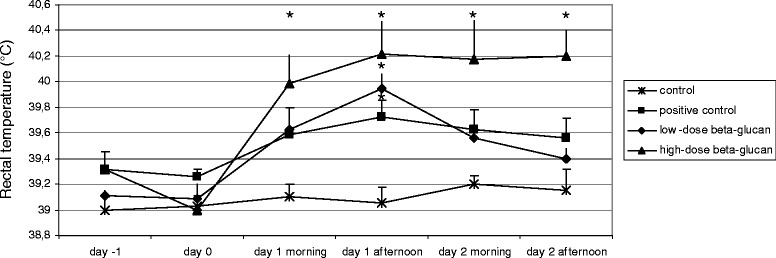
**Mean rectal temperature of rabbits before and after physiological** **(in.)** ***P. multocida*** **challenge.** Day 0 indicates the day of the challenge. Significant differences between groups are indicated with asterisk (*p <0.05). Data are shown as means + SEM.

**Figure 2:**
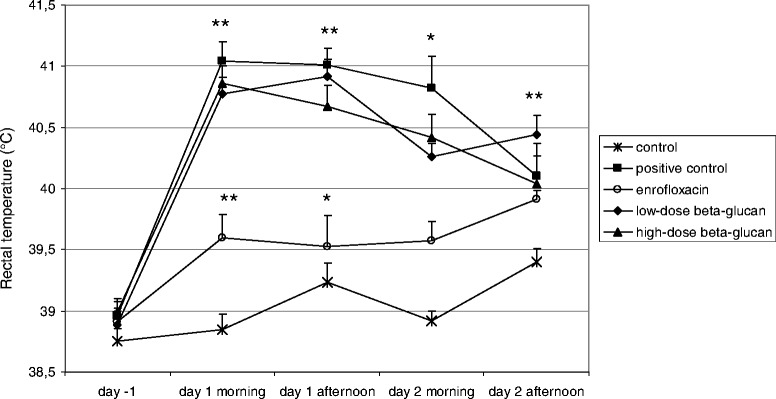
**Mean rectal temperature of rabbits before and after severe** **(in. and im.)** ***P. multocida*** **challenge.** Day −1 indicates the day before the challenge. Significant differences between groups are indicated with asterisks (*p <0.05; **p <0.01). Data are shown as means + SEM.

In the physiological model, at 24 hours after physiological challenge (in. only), infected animals developed conjunctivitis and significantly increased rectal temperature (p ≤0.05), clinical score ranged between 1 and 2. Animals exposed to supra-physiological challenge (in. and im.) had, in addition to the above, nasal discharge and respiratory murmur, clinical score ranged between 3 and 5. Rectal temperature significantly increased 24 hours after both challenges (p ≤0.05) and (p ≤0.01) (Figures 1 and 2). The control rabbits showed no clinical signs.

**Figure 3:**
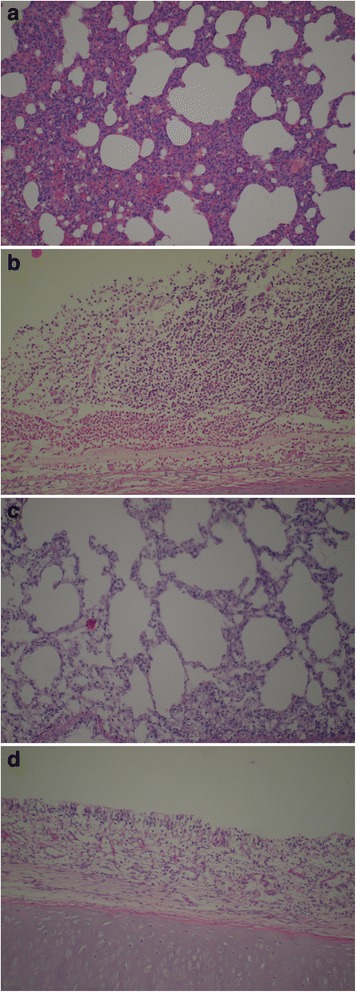
**Representative photomicrographs of** **(a,** **c)** **lung tissue and** **(b** **,** **d)** **nasal mucosa from rabbits infected intranasally with** ***P. multocida.*** **(a)** Lung from the positive control (untreated) animals showing interstitial inflammatory reaction, and **(c)** normal lung from the β-glucan treated animals. **(b)** Nasal mucosa from the positive control (untreated) animals showing epithelial necrosis and intensive heterophil granulocyte infiltration with submucosal edema. **(d)** Normal nasal mucosa from the β-glucan treated animals. Hematoxylin and eosin stain; magnification, 100×.

In the animals exposed to physiological (in.) challenge, autopsy findings were relatively minor (see Additional file 1); nasal mucosa showed epithelial damage and inflammatory infiltration and the lungs were affected by interstitial pneumonia (Figure 3A-B), but macroscopic lesions did not develop. Tissues collected from the control and β-glucan treated groups were healthy and intact (Figure 3C-D). *P. multocida* was cultured back from all challenged rabbits nasal cavities. In the supra-physiological model (rabbits infected in. and im.), autopsy revealed congested heart accompanied with enlarged and congested blood vessels, necrotic foci in the liver, brown peritoneum, congested friable kidneys, and dark brown spleen. Trachea, lungs, and heart were congested, hyperemic and filled with blood. The lungs showed peribronchitis, severely congested vessels with vasculitis, marked alveolar collapse, diffused interstitial inflammatory reaction and intra-alveolar tissue destruction. Positive control and β-glucan animals showed similar pathology. The enrofloxacin treated group had milder symptoms; interstitial pneumonia and *Pasteurella* septicaemia occurred but necrotic lesions and macroscopical signs were not seen. *P. multocida* were cultured back from all inflammatory foci.

**Figure 4:**
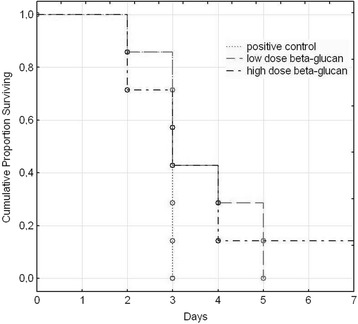
**Impact of β**-**glucan treatements on rabbits infected intranasally and intramuscularly with** ***P. multocida.*** Rabbits were received methylcellulose solution (positive controls, n = 7) and either low-dose (n = 7) or high-dose (n = 7) β-glucan (5 or 50 mg/kg bw.). Curves were calculated by Kaplan-Meier method and Cox’s F test was used for comparisons between treatments. Positive control x low-dose β-glucan (F = 2.306, p = 0.065), positive control x high-dose β-glucan (F = 2.344, p = 0.065), positive control x both β-glucan treatments (F = 2.408, p = 0.044).

β-glucan treatment did not reduce the numbers of deaths in the supra-physiological model but did postpone death by several days. Either low-dose or high-dose β-glucan treatment marginally delayed death but the delay was statistically significant when both β-glucan treatments were compared to the positive control group together (Figure 4). In field conditions, this extended window of opportunity for antibiotic intervention would have considerable implications for veterinary management.

All ten *P. multocida* isolates administered to the rabbits were shown to be sensitive to enrofloxacin in the disk diffusion test. The inhibition zone diameters were between 21–25 mm. All ten *P. multocida* isolates harboured the species-specific gene sequence (*kmt*), porin protein encoding gene (*ompH*), hemoglobin binding protein encoding gene (*hgbA*), filamentous hemagglutinin encoding gene (*pfhA*), neuraminidase gene (*nanH*) and 8 of 10 isolates were contained the type 4 fimbriae gene (*ptfA*). 8 of ten isolates were positive for *capA* capsule biosynthesis gene.

## Discussion

In rabbit husbandry, good environmental control is critical in reducing the burden of infection. Common symptoms including nasal discharge, sneezing and conjunctivitis are most frequently due to *Pasteurella multocida*, which is also the major cause of morbidity and mortality. *P. multocida* may also cause abscesses in subcutaneous and other sites, torticollis, circling due to infection of the inner ear and septicaemia [13].

Fluoroquinolones such as enrofloxacin are concentration-dependent bactericides with activity against many pathogens including *P. multocida*. Tissue levels two to three times higher than that found in the serum have been demonstrated in various species, and the fluoroquinolones are relatively safe in rabbits [16], but questions regarding effectiveness remain. For example, enrofloxacin (5 mg/kg bw.) given orally to rabbit does stopped transmission of *Pasteurella* to their young but failed to eliminate infection from the mother rabbits themselves [17]. Enrofloxacin given at 5 mg/kg im. eliminated *P. multocida* from nearly all adult rabbits but in rabbits given enroflaxacin orally, although the nasal flush cultures were negative, the necropsy cultures from nasopharynx, cavity, tympanic cavity, and lung were all positive [17]. These findings correspond with our results, where animals treated with 10 mg/kg enrofloxacin p.o. showed less serious clinical signs and necropsy alterations, but interstitial pneumonia still occured.

It is generally acknowledged that Pasteurellosis in rabbits should be managed rather than treated, but the failure of oral enrofloxacin to eradicate *P. multocida* and the subsequent risk of resistance has led to regulatory pressure to phase these drugs out and seek alternatives. The (1–3), (1–6) β-glucans are prime candidates. Numerous studies have shown that these natural compounds exhibit anti-infective and anti-tumour properties, mediated by immuno-priming. In immuno-priming, β-glucans bind to the CR-3 receptor present on innate immune cells resulting in enhanced chemotaxis, phagocytosis and microbial killing [18]. This has been shown to confer protection against many pathogens including anthrax, a lethal disease that affects animals and humans. In one study conducted in association with the Canadian Department of Defence [19], orally administered Immivet® 3–6 (2 and 20 mg/kg body weight) given for eight days prior to innoculation with *Bacillus anthracis* provided almost complete protection against infection over the 10-day post-exposure test period.

The result of our study indicates that clinical disease caused by physiological exposure to *P. multocida* can be prevented with oral β-glucans. This protective action should enable reduced antibiotic use in livestock and pets, leading to improved food safety and reduced spread of resistance. The immuno-priming activity of the (1–3), (1–6) β-glucans when given together with antibiotics has been shown to increase the effectiveness of the antibiotics [20]. This will be evaluated in further trials.

## Conclusions

Supra-physiological exposure to *P. multocida* (in. and im. innoculation) is lethal in rabbits. Physiological exposure (in. only) creates a milder and non-lethal infection. We used *Pasteurella* isolates with serious virulence-associated genes (*ompH*, *hgbA*, *pfhA*, *nanH*, *ptfA*). After supra-physiological exposure, only the enrofloxacin treatment was clinically effective, and even the antibiotic treated group suffered from septicaemia. β-glucan treatment did not prevent death, although it delayed death considerably compared to controls. β-glucan was, however, highly effective in protecting against physiological exposure to *P. multocida*, preventing any histological damage. Our results suggest that β-glucan feeding should be preventive against clinical Pasteurellosis in rabbits.

## Additional file

Additional file 1
**Evaluation of the histological sections of lungs, spleen, liver, kidneys, and nasal mucosa derived from the intranasally challenged (** ***P. multocida*** **) rabbits.** The +/− indicates the presence/absence of histopathological alterations, respectively.

## Abbreviations

bw.: bodyweight
CFU: colony forming unit
CR-3: complement receptor 3
im.: intramuscular
in.: intranasal
ip.: intraperitoneal
MÁB: Munkahelyi Állatjóléti Bizottság (Local Institutional Animal Care Committee)
pi.: postinfection
*P.*: *Pasteurella*
p.o.: *per os*
SEM: standard error of mean
