# Supplementary material for: Alternative treatment of serious and mild Pasteurella multocida infection in New Zealand White rabbits
Source: BMC Vet Res. 2014 Nov 25;10:276. doi: 10.1186/s12917-014-0276-6 (PMC4248437; doi:10.1186/s12917-014-0276-6)
Supplement: Additional file 1 — Evaluation of the histological sections of lungs, spleen, liver, kidneys, and nasal mucosa derived from the intranasally challenged ( P. multocida ) rabbits. The +/− indicates the presence/absence of histopathological alterations, respectively. [file 12917_2014_276_MOESM1_ESM.zip › 2735398191309432_add1.rtf]

Histological results of the intranasally challenged rabbit experiment

	Nasal mucosa	Lungs	Spleen	Liver	Kidneys	
Control	-	-	-	-	-	
Control	-	-	-	-	-	
Control	-	-	-	-	-	
Control	-	-	-	-	-	
Control	-	-	-	-	-	
Control	-	-	-	-	-	
Control	-	-	-	-	-	
Positive control	+	+	-	-	-	
Positive control	+	-	-	-	-	
Positive control	+	+	-	-	-	
Positive control	+	+	-	-	-	
Positive control	+	-	-	-	-	
Positive control	+	-	-	-	-	
Positive control	+	-	-	-	-	
Low dose beta-glucan	-	-	-	-	-	
Low dose beta-glucan	-	-	-	-	-	
Low dose beta-glucan	-	-	-	-	-	
Low dose beta-glucan	-	-	-	-	-	
Low dose beta-glucan	-	-	-	-	-	
Low dose beta-glucan	-	-	-	-	-	
Low dose beta-glucan	-	-	-	-	-	
High dose beta-glucan	-	-	-	-	-	
High dose beta-glucan	-	-	-	-	-	
High dose beta-glucan	-	-	-	-	-	
High dose beta-glucan	-	-	-	-	-	
High dose beta-glucan	-	-	-	-	-	
High dose beta-glucan	-	-	-	-	-	
High dose beta-glucan	-	-	-	-	-	
(+/-) presence/absence of histopathological alterations
